# The oral health status, behaviours and knowledge of patients with cardiovascular disease in Sydney Australia: a cross-sectional survey

**DOI:** 10.1186/s12903-018-0697-x

**Published:** 2019-01-11

**Authors:** Paula Sanchez, Bronwyn Everett, Yenna Salamonson, Julie Redfern, Shilpi Ajwani, Sameer Bhole, Joshua Bishop, Karen Lintern, Samantha Nolan, Rohan Rajaratnam, Maria Sheehan, Fiona Skarligos, Lissa Spencer, Ravi Srinivas, Ajesh George

**Affiliations:** 1 School of Nursing and Midwifery Western Sydney University, Centre for Oral Health Outcomes & Research Translation (COHORT), Ingham Institute Applied Medical Research, Liverpool Campus, Locked Bag 1797, Penrith, NSW 2751 Australia; 2 School of Nursing and Midwifery Western Sydney University, Ingham Institute Applied Medical Research, Centre for Applied Nursing Research (CANR), Parramatta South Campus, Locked Bag 1797, Penrith, NSW 2751 Australia; 3 School of Nursing and Midwifery Western Sydney University, Ingham Institute Applied Medical Research, Centre for Applied Nursing Research (CANR). Campbelltown Campus, Locked Bag 1797, Penrith, NSW 2751 Australia; 4 Westmead Applied Research Centre, Faculty of Medicine and Health University of Sydney, PO Box 154, Westmead, NSW 2154 Australia; 5 Sydney Local Health District, Oral Health Services, Sydney Dental Hospital, University of Sydney/ Sydney Research, 2 Chalmers Street, Surry Hills, NSW 2010 Australia; 6 Balmain Hospital, 29 Booth, St Balmain, NSW 2041 Australia; 7 South Western Sydney Local Health District, Cardiac Ambulatory Services, Liverpool Hospital, 7103 Locked Bag 7103, Liverpool BC, NSW 2170 Australia; 8 Cardiac Ambulatory Services Cardiac Rehabilitation, Royal Prince Alfred Hospital & Balmain Hospital, 29 Booth St Balmain, Camperdown, NSW 2041 Australia; 9 South Western Sydney Local Health District, Locked Bag 7103, BC1871, Liverpool, NSW 2170 Australia; 10 South Western Sydney Local Health District, Cardiac Rehabilitation and Chronic Cardiac Care, Fairfield Hospital, Polding St & Prairie Vale Rd, Prairiewood, NSW 2176 Australia; 11 Cardiac Ambulatory Services, Cardiac Rehabilitation, Royal Prince Alfred Hospital, 50 Missenden Rd, Camperdown, NSW 2050 Australia; 12 Chronic Disease Rehabilitation, Royal Prince Alfred Hospital, 50 Missenden Rd, Camperdown, NSW 2050 Australia; 13 South Western Sydney Local Health District Oral Health Services, Centre for Oral Health Outcomes & Research Translation (COHORT), Western Sydney University, University of Sydney, Ingham Institute Applied Medical Research, 59 Cumberland Rd, Ingleburn, NSW 2565 Australia; 14 Centre of Oral Health Outcomes & Research Translation (COHORT), Western Sydney University, South Western Sydney Local Health District, University of Sydney, Ingham Institute Applied Medical Research, Translational Health Research Institute, Level 3, 1 Campbell, St Liverpool, NSW 2170 Australia

**Keywords:** Cardiovascular disease, Oral health behaviours, Oral health knowledge, Oral health status, Periodontal disease

## Abstract

**Background:**

Periodontal disease is a risk factor for atherosclerotic cardiovascular disease and it is recommended internationally that patients with cardiovascular disease should engage in preventative oral health practices and attend regular dental care visits. This study aimed to explore the oral health status, behaviours and knowledge of patients with cardiovascular disease.

**Methods:**

A cross-sectional questionnaire containing 31 items was administered to patients with cardiovascular disease from cardiac rehabilitation and outpatient clinics in Sydney Australia in 2016–2017.

**Results:**

Of the 318 patients surveyed, 81.1% reported having at least one oral health problem. Over a third (41.2%) of participants had not seen a dentist in the preceding 12 months and 10.7% had received any oral healthcare information in the cardiac setting. Those with valvular conditions were more likely to have received information compared to those with other cardiovascular conditions (40.6% versus 7.4%, *p* < 0.001). Only half of the participants had adequate oral health knowledge.

**Conclusions:**

Despite a high incidence of reported oral health problems, many patients lacked knowledge about oral health, were not receiving oral health information from cardiac care providers and had difficulty accessing dental services. Further research is needed to develop oral health strategies in this area.

**Electronic supplementary material:**

The online version of this article (10.1186/s12903-018-0697-x) contains supplementary material, which is available to authorized users.

## Background

There is an association between periodontal disease and the prevalence of cardiovascular disease (CVD) with growing evidence suggesting that periodontal disease is a risk factor for atherosclerotic cardiovascular disease (ASCVD) [1–3]. Periodontal disease or periodontitis is a chronic inflammatory disease affecting the tooth supporting tissues and bone. It is caused by a host response against bacterial infection involving the oral cavity and dental plaque leading to tooth loss [4]. Periodontitis contributes to the global burden of chronic oral diseases and is a major public health problem worldwide [5–8]. In Australia, periodontitis is the fifth most common health challenge with a prevalence of 23% for moderate to severe types of the disease with higher incidence in males (26.8% compared with 19.0%) and in older people (53.4% at age > 65) [9].

It has been suggested that systemic inflammation triggered by periodontitis is linked to ASCVD. Bacteria that cause periodontitis enter the blood stream leading to a systemic host-mediated inflammatory response, which contributes to atheroma formation, maturation and exacerbation [2]. Exacerbating the situation is the common adverse effects on the oral cavity of a number of medications used for the treatment of ASCVD including xerostomia or dry mouth, hypo salivation, and taste disturbances [10, 11]. There is also increasing evidence that periodontal treatment could reduce systemic inflammation [12–14] although its effectiveness in improving long term CVD outcomes is still uncertain [15]. Nevertheless, international consensus statements recognise the relevance of the association between periodontal disease and ASCVD and recommend preventative oral health approaches to be adopted in cardiac care settings [2, 7, 16] and that all patients with CVD engage in preventative oral health practices and attend regular dental care to reduce cardiovascular risks [16–18].

Despite these recommendations, research reports about the oral health status and practices of adults with CVD are limited. In a cross-sectional study involving 150 adult Iranian patients with heart disease, the authors found that oral health practices were poor and patients’ knowledge was moderate [19]. Most studies in this area have focused on children with cardiac diseases and their parents/caregivers. These studies showed poor oral health status among the children and inadequate oral health knowledge and practices among their parents [20–23]. Overall, research and understanding about the oral health status, behaviours and knowledge of adults with CVD is limited, particularly in Australia. Therefore, the aim of this study was to describe the self-reported oral health status, behaviours and knowledge of adult patients with CVD in the Australian setting. Research questions were:

1. What is the prevalence of self-reported oral health problems among adult patients with CVD?
2. What are the reported oral health behaviours of patients with CVD?
3. What is the perceived level of knowledge regarding oral health and CVD in these patients?

## Methods

### Design

A quantitative cross-sectional questionnaire of patients with CVD was undertaken.

### Sampling and setting

The convenience sample consisted of 318 patients who were attending outpatient cardiology services in Sydney Australia between December 2016 and March 2017. Outpatient services included four cardiac rehabilitation sites, two public cardiology clinics and one private clinic in both affluent and disadvantaged locations in the Sydney region. Recruitment from these sites ensured patients with varied CVD conditions and socio-economic status were included in the sample. Inclusion criteria were people: a) with a diagnosis of CVD; b) over 18 years old; and c) who were English-speaking. Exclusion criteria were those people with limited English language and those who did not have an interpreter or a family member present who could interpret the questionnaire at the time of data collection. Ethical approval was obtained by the Sydney and South Western Sydney Local Health District Human Research Ethics Committees (LNR/16/LPOOL/499).

### Data collection procedure

Flyers advertising the study were distributed across waiting rooms of the study sites. Interested participants were directed to a dental stall which was set up at in the waiting rooms. An experienced researcher and dental professional explained the purpose of the project to patients who approached the stall. If patients indicated interest, they were provided with a self-administered questionnaire to complete while waiting for their medical appointment. Participation was voluntary and oral health information and dental products were provided to patients regardless of study participation. Written consent was obtained from all participants. Completion of the questionnaire took between 10 and 15 min.

### The questionnaire tool

The study questionnaire was adapted from an existing validated instrument which was developed to assess the oral health status, behaviour and knowledge in pregnant women [24]. Only some items were revised for the study population. Item generation for the questionnaire was guided by Andersen’s model [25] to assess the factors influencing access to dental care among people with CVD. The questionnaire contained items relating to respondents’ reported oral health status, oral health care behaviours and perceptions, their confidence in dental self-care, oral health knowledge and beliefs, information received about oral health since cardiac diagnosis, and social and family support. Socio-demographic data was also collected. Some additional knowledge items included oral health related side effects of commonly prescribed cardiac medications [10, 11, 26]. The questionnaire also included items reported elsewhere including barriers to seeking dental care [24]. To establish face and content validity, the questionnaire was reviewed by an expert panel consisting of clinicians, academics and educators in the field of dentistry, cardiology and physiotherapy and their agreement of the survey items were sought through qualitative feedback [27]. Minor revision of items was undertaken based on feedback received. The questionnaire was then piloted with six patients with CVD (not participating in the study) to ensure readability and relevance [28] and further refined. An additional file shows the questionnaire tool completed by participants of the study [see Additional file 1].

### Sample size

The oral health behaviour - uptake of dental services by people with CVD, was used for the sample size estimation. However, due to the lack of this information in Australia, the uptake of oral health services among another vulnerable population (pregnant women) who are impacted by poor oral health (pregnant women) in Australia and patients with CVD internationally was used to inform the sample size. In a recent Australian study 30% of pregnant women were reported to have visited the dentist in the last 12 months even when they had dental problems [29]. Given that people with CVD were older and thus, more likely to have dental problems, we estimated that 40% of responders would have had a dental visit in the preceding 12 months. Further, one international study showed 38% of patients with CVD had seen a dentist regularly [30]. Hence the sample size was calculated as 250. Allowing for 20% missing data, a sample size of 300 was required.

### Data analysis

The data was analysed using Statistical Package for the Social Sciences (SPSS) Version 24 software [31]. Descriptive statistics such as mean and standard deviation for continuous variables and frequency and percentage for categorical variables were calculated and tabulated. Median and inter quartile range (IQR) were calculated for non-normally distributed data. Bivariate (Pearson) analysis was undertaken to determine the correlation between the participant’s socio demographics, self-reported oral health status and behaviour characteristics with their level of oral health knowledge. The total score was aggregated.

## Results

### Demographic characteristics

A total of 318 participants completed the study. Sixty percent of the participants were males and the age of participants ranged from 18 to 94 years (mean 63.7, *SD* 14.5). More than half of the participants were born outside Australia (57.9%) and spoke a language other than English (58.2%). Two thirds were not working at the time of the questionnaire (67.8%) and almost half (49%) had low combined income of AUS$ < 40,000. Most participants had secondary or tertiary education (85.8%) and two thirds were living with a partner (67.4%). In relation to their cardiac diagnosis, coronary artery disease was the most common CVD condition among the participants (73.6%) with others (not mutually exclusive) suffering from hypertension, arrhythmias, heart failure and valvular conditions [32]. Coronary artery disease was more prevalent in males compared to females (64.1%, *n* = 150 versus 54.9%, *n* = 84). Almost half of the participants (45.4%) were eligible for public or free dental service either via holding a health care card or being a member of the defence force (Table 1).

**Table 1:** Sociodemographic and clinical characteristics of people with cardiovascular disease (*n* = 318)

Variables	Frequency (%)
Age, mean (*SD*) in years	
Gender	
Male	191 (60.1)
Country of birth	
Australia	134 (42.1)
Marital status^a^	
Living with a partner	213 (67.4)
Language spoken at home	
English	133 (41.8)
Employment status^a^	
Not working	213 (67.8)
Highest educational achievement^a^	
Up to primary schooling	45 (14.2)
Secondary schooling	118 (37.2)
Tertiary studies	154 (48.6)
Income^a^	
< $40,000	157 (49.4)
$40,000 to $79,999	62 (19.5)
$80,000 to $120,000	34 (10.7)
> $120,000	25 (7.9)
Preferred not to answer	40 (12.6)
Private Health Insurance^a^	
Yes	118 (37.2)
Eligible for public or free dental service^a^	
Yes	144 (45.4)
Cardiovascular condition(s) (not mutually exclusive)	
Coronary artery disease	234 (73.6)
Hypertension	109 (34.3)
Arrhythmias	94 (29.6)
Heart failure	43 (13.5)
Valvular condition	32 (10.1)
Years since cardiac diagnosis, mean (IQR) Range	7.80 (11.0) 0–50
Other co-morbidities as per ICD-10 [31] (*n* = 239)	
Circulatory system (hypertension/vascular disorders)	75 (31.4)
Endocrinology/metabolic (diabetes/thyroid/renal failure)	74 (31.0)
Musculoskeletal/connective tissue (arthritis/osteoporosis/Lupus)	30 (12.6)
Neoplasms (prostate/breast other cancers)	14 (5.9)
Others (respiratory/digestive/ears/eyes/mental health)	46 (19.2)

^a^Missing data (range 1–4)

### Self-reported oral health status and behaviour

More than half of the participants with CVD claimed to having a good to excellent oral health status (56.1%) but the majority (81.8%) reported having at least one oral health problem at the time of the study, with over a third (41.8%) admitting that their oral health problem affected what they ate. Some of the main concerns reported (multiple responses) included teeth that did not look right (broken, crooked or discoloured) (41.0%), dry mouth (40.1%) and sensitive teeth (32.5%). Other problems reported included missing teeth and dentures that did not fit (11.6%). There was no significant difference between participants reporting oral health problems and their socio-economic status. However, those who had private health insurance, lower income, had been diagnosed for more than four years and were older (> 65 years), had more self-reported problems. The majority of respondents (83.4%) noted that their dental health was important compared to their overall health. Many participants reported cleaning their teeth or dentures twice or more times per day (60.4%). Fluoride toothpaste was used by the majority of participants (90.9%) but only a third (34.6%) used dental floss or other aids to clean between their teeth (Table 2). Participants had a high confidence score in managing their oral health with a mean of 12 out of the maximum rating of 15.

**Table 2:** Oral health status and behaviour of people with cardiovascular disease (*n* = 318)

Variables	Frequency (%)
Oral health status^a^	
Excellent	15 (4.7)
Very good	60 (18.9)
Good	103 (32.5)
Fair	99 (31.2)
Poor	40 (12.6)
Self-reported oral health problems	
One problem or more	260 (81.8)
Type of oral health problems^b^	
Teeth that don’t look right (broken, crooked, discoloured)	130 (41.0)
Dry mouth	127 (40.1)
Sensitivity	103 (32.5)
Cavities	93 (29.3)
Toothache	65 (20.5)
Bleeding/swollen/painful gums	58 (18.3)
Loose teeth	46 (14.5)
Other problems	37 (11.6)
Dental problem affects what you eat	
Yes	133 (41.8)
Use of partial or full dentures	
Yes	16 (5.0)
Importance of oral health compared to overall health^a^	
Low importance (0–4)	23 (7.3)
Neutral (5)	29 (9.2)
Important to extremely important (6–10)	262 (83.4)
How often do you brush your teeth/dentures	
Few times a week	14 (4.4)
Less than once per day	6 (1.9)
Once a day	103 (32.4)
Twice or more times a day	192 (60.4)
Never	3 (0.9)
Oral hygiene products used^b^	
Fluoride toothpaste	289 (90.9)
Dental floss or other aids	110 (34.6)
Mouthwash	93 (29.2)
Sugar free chewing gum	36 (11.3)
None	10 (3.1)
Seen a dentist in the last 12 months	
Yes	187 (58.8)
When was your last dental visit	
< 1 year	187 (58.8)
> 1 year to 2 years	43 (13.5)
> 2 years to 5 years	43 (13.5)
> 5 years	39 (12.3)
Don’t know	6 (1.8)
Where do you most often see the dentist^a^	
Private clinic	222 (70.3)
Public clinic or hospital (government funded)	69 (21.8)
Other	16 (5.1)
Don’t know	9 (2.8)
Received information about oral health care since diagnosis^a^	
Yes	34 (10.7)

^a^Missing data (range 1–4); ^b^Multiple responses

Over half of the respondents had visited a dentist in the last 12 months (58.8%), and over one quarter of the participants reported their last dental visit was greater than two years prior to completing the questionnaire. The main dental services used by participants were private dentists (70.3%) compared to 21.8% who attended the public dental service. Only 10.7% received any information since their cardiac diagnosis (Table 2). Of all the participants with valvular conditions (*n* = 32) less than half (40.6% *n* = 13) reported receiving any oral health information but overall they were more likely to have received information compared to those with other cardiovascular conditions (40.6% versus 7.4%, *p* < 0.001).

### Oral health knowledge

**Fig. 1:**
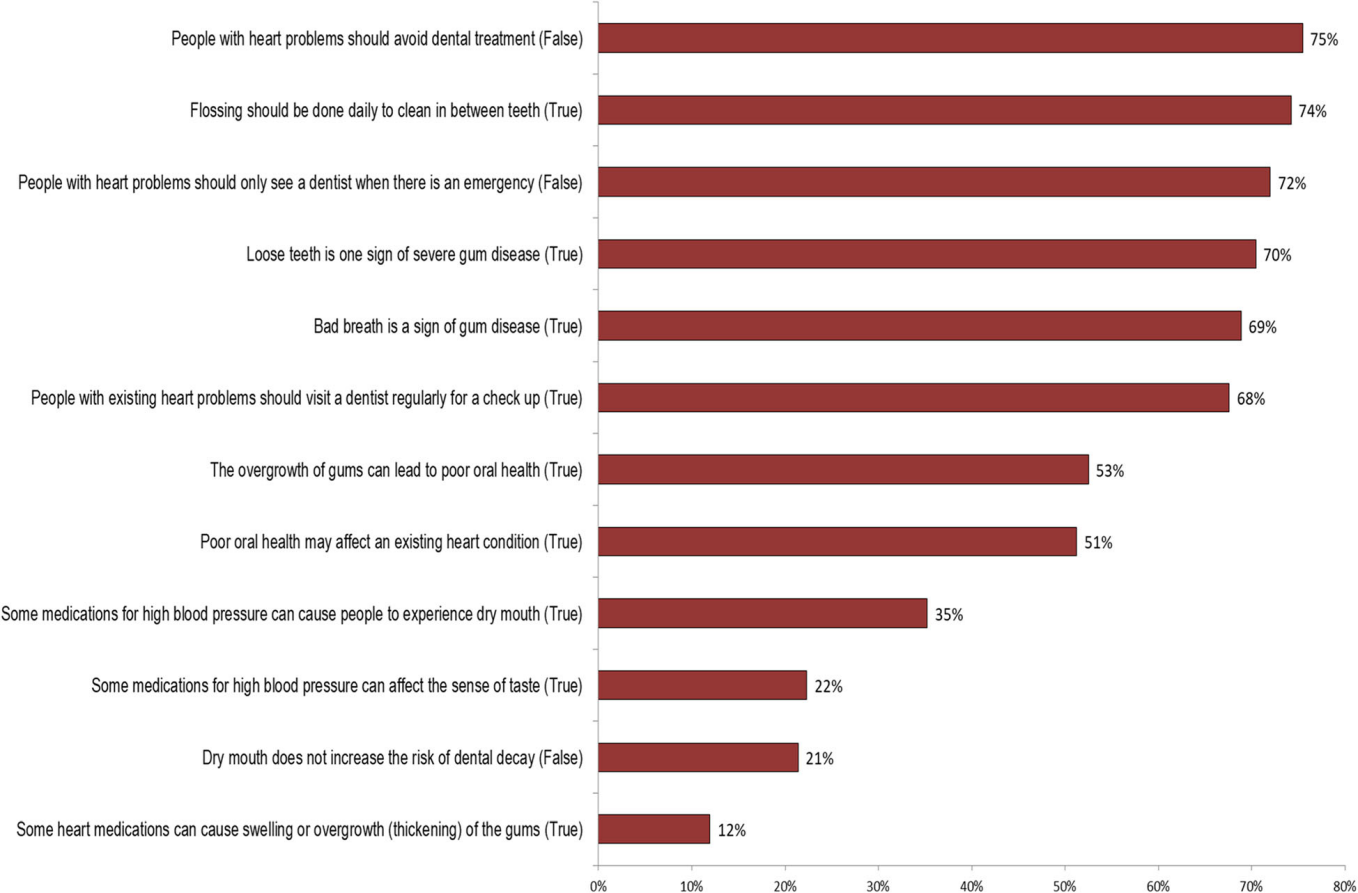
Percentage of correct questionnaire responses from participants (*n* = 318)

The mean correct responses for the 12 knowledge items was 6.2% (median 6, *SD* 2.8) indicating that only half of the participants had adequate oral health care knowledge. No significant difference in the oral health knowledge was observed among participants based on their socio demographic factors. However, the Pearson correlation revealed that there was a weak correlation (*r* = 0.115, *p* = 0.041) between the oral health knowledge and the participant’s level of education. Similarly, a weak correlation (*r* = 0.121, *p* = 0.031) was observed between the participant’s knowledge and whether they received oral health information in the cardiac setting. Areas of high awareness were about the need for regular dental visits when having a cardiac condition, regular flossing and common signs of gum disease (loose teeth, bad breath) (68–75% correct responses). Areas where knowledge was poor included the effect of cardiac medications on dry mouth and gum overgrowth as well as the relationship between poor oral health and an existing cardiac condition (12–53% correct responses) (Fig. 1).

## Discussion

The main purpose of this study was to gain insight into the oral health status, behaviour and knowledge of people with CVD.

### Demographic characteristics of sample

The study sample reflected an even spread of participants from two ethnically and financially diverse areas in Sydney. Around half the participants in this study were from a lower socioeconomic group, were born overseas and spoke a language other than English at home. In Australia people with low income (AUS$ < 61,035) are entitled to cheaper public transport, medicines and are eligible to public oral health services [33, 34]. Forty-five percent of participants in the study were eligible for public dental service which is consistent with population data in New South Wales (47%) [34]. Half of the participants completed year 12 or equivalent studies which is similar to population data for the study setting where 45.6% participants reported having secondary education or less [35, 36]. The study participants had similar characteristics with the Australian population of people with CVD in relation to a higher prevalence of males (6.9% compared to 4.6% for women) and similar types of cardiac conditions including coronary heart disease, heart failure and diseases of arteries/arterioles/capillaries [37].

### Self-reported oral health status and behaviour

In the study sample the number of participants who reported having at least one oral health problem (81.8%) was of concern. While there is no specific data on the oral health status of patients with CVD, a report on oral health and dental care in Australia found 22.9% of dentate people aged 15 and over had moderate or severe periodontal disease [38]. Prevalence was higher in older adults; in those aged 45–64, rates were 35.3% increasing to 53.4% in those aged 65 and over [38]. Given atherosclerotic cardiovascular disease is associated with aging [1–3] it is likely that many of those with periodontal disease also had ASCVD.

In the current study 41.8% of participants reported their oral health problem affected what they ate which is higher than Australian data (23% of people aged over 65 years) [38]. Apart from the impact on quality of life [39] restricting foods because of pain and difficulty chewing could result in reduced intake of recommended hard foods such as fruits and vegetables which are essential for the prevention and management of CVD [40].

Overall, compared with the Australian general population the incidence of self-reported problems in the study sample was higher. One of the main reasons for this could be the poor socioeconomic status of nearly half the participants and their limited access to private health insurance. It is well documented in Australia that people with reduced income (<AUS$60,000) and those not having private health insurance are more likely to have oral health problems such as untreated tooth decay and missing teeth, and the incidence is higher in older adults > 45 years [38]. Another contributing factor could be that patients in the sample had multiple co-morbidities such as endocrinology and metabolic disorders, including diabetes, which is associated with a high incidence of periodontal disease [41]. Furthermore, even though data on medications was not collected, it is likely that the participants in the study were taking a variety of cardiac medications putting them at a higher risk for gingival and periodontal pathology [10, 11, 42]. A common issue associated with calcium channel blocker agents is gingival enlargement (hyperplasia) which can lead to tooth loss due to increased incidence of periodontal disease [43, 44]. Beta-blockers are associated with mucosal lesions [45]. The problems identified in the study sample reflect the type and incidence of oral health problems linked to cardiac medications. In the study only 5% of participants used partial or full dentures but this can also be associated with problems such as denture-associated stomatitis and other mucosal lesions with systemic consequence [46].

In Australia there is no data on the oral health care uptake for people with CVD but oral health seeking behaviour of Australian general population from 2013 reported 55.2% of adults aged 22–44 visited the dentist in the previous 12 months with the number increasing as people were older, (63.8% for age 45–64 and 70.1% for age > 65) [38]. The uptake of dental services by the study participants in the previous 12 months was lower for the same age groups but the age trend was similar, increasing by age. To our knowledge only one study internationally has explored the uptake of dental services among patients with CVD and the reported rate (38%) was lower than this study (59%) [30]. Participants had a high confidence level in oral self-care (mean 13, SD 2.2, range 2–15) and it was encouraging to see that two thirds reported good oral hygiene habits such as regular tooth brushing and flossing. This is particularly important as studies show that these habits are associated with reduced risk factors for CVD [47–49].

Of concern however was that only 10.7% of participants reported receiving any oral health information since being diagnosed with a cardiac condition. Even in participants with a valvular condition who are required to have dental clearance prior to surgery [50, 51], less than half reported receiving information. People who require dental clearance should be provided with oral health information. These findings confirm earlier qualitative reports [52] and strongly suggest that people with cardiac conditions are not receiving any oral health information in the cardiac setting. It is also possible that people with CVD who had limited knowledge about the potential impact of poor oral health on cardiac condition (51%) may not have prioritised any oral health information provided. Recent studies have also identified potential barriers that could be contributing to this problem which include lack of oral health awareness by patients and cardiac care clinicians, lack of training by clinicians, limited oral health resources on CVD, as well as time constraints among cardiac care providers [15, 24].

### Oral health knowledge

It is worrying that only half the participants knew the impact of oral health and ASCVD. Unfortunately, there is no comparable population data about the oral health knowledge of people with CVD in Australia. Looking into other populations in Australia it has been shown that a number of pregnant women (53%) are unaware of the association between poor oral maternal health and pregnancy outcomes [29]. Similar observations have been reported internationally though, with studies showing a lack of knowledge around the relationship between oral health and heart conditions among caretakers of children with heart diseases (49–90%) as well as patients with CVD (42%) [19–23]. The poor knowledge observed in this study could be linked to the lack of oral health resources and information provided in the cardiac setting. Despite current national oral health plans, there is limited availability of evidence based oral health promotional resources in the cardiac setting [18, 53]. It appears current policies and guidelines are not meeting the oral health needs of people with cardiovascular conditions [53]. This is supported by a recent scoping review which found that in Australia, as well as internationally, people with CVD are not offered routine oral health education, assessment and prompt referral when treatment may be required [15].

### Limitations

Data reported in this study is based on self-reported information of patients who sought cardiac health service from two local health areas in the Sydney region, so this limits the ability to determine the actual prevalence of oral health problems among this population. However, the two areas had a diverse population and hence we have a reasonable level of generalisability. In addition, even though the use of convenience sampling limits the generalisability (external validity) of the findings of this study, we do not believe convenience sampling poses a significant threat to the internal validity of this study, as this sampling method has been mathematically shown to be as accurate as random sampling when recruiting patients attending clinics for their health appointment [54]. Another study limitation is that some people with limited English language may have been excluded from the study even though all attempts were made to include those using professional interpreters or family members. As such the findings may not be generalizable to culturally and linguistically diverse populations with CVD who have oral health problems. Due to the lack of existing tools to explore this aspect of cardiac care, the questionnaire used for this study was not validated and hence further research is needed to confirm the study findings. In summary, the scope of this paper was to present descriptive data related to knowledge, oral health status and behaviour. Multivariate statistical analysis examining the predictors of accessing oral health care for this population has been undertaken and reported elsewhere [55]. Despite these limitations the findings do provide valuable insight into this under researched area and pave the way for future validation studies to be undertaken using the study questionnaire.

### Recommendations

To address some of the issues identified in this study a multidimensional approach is needed. The development and incorporation of evidence-based oral health resources for cardiac care clinicians and patients are needed in the cardiac setting. The availability of information and resources influence behavioural choices therefore if oral health is promoted among people with cardiac conditions perhaps they would be more knowledgeable and proactive regarding their oral health. It is also essential to identify barriers that may be impeding access to oral health care for people with CVD and explore their perceptions of incorporating oral health in the cardiac setting. A number of other factors need to be considered when developing strategies in this area and include the affordability and accessibility of dental care for people with CVD. Future related studies should also consider obtaining information regarding cardiac medications used by people with CVD due to the common adverse effects it can have on the oral cavity.

## Conclusions

There is limited data concerning the oral health status and behaviour of people with CVD. Our findings suggest that people with CVD may have a high prevalence of self-reported oral health problems and poor knowledge about the importance of oral health particularly about the link between periodontal disease and CVD and the effect that some cardiac medications may have on oral health. Results of this study also suggest that people with CVD may not be receiving adequate oral health information from cardiac care providers after diagnosis. Exacerbating this situation could be the limited uptake of dental services and access to affordable dental care among people with CVD. Further research is warranted to confirm the study findings and the need for strategies to improve the oral health of people with CVD.

## Additional file

Additional file 1: Participant questionnaire. This file includes the questionnaire completed by participants in the study (PDF 269 kb)

## Abbreviations

ASCVD: Atherosclerotic cardiovascular disease
CVD: Cardiovascular disease
ICD-10: International Statistical Classification of Diseases and Related Health Problems 10th Revision
IQR: Inter quartile range
SPSS: Statistical Package for the Social Sciences
