# Supplementary material for: The oral health status, behaviours and knowledge of patients with cardiovascular disease in Sydney Australia: a cross-sectional survey
Source: BMC Oral Health. 2019 Jan 11;19:12. doi: 10.1186/s12903-018-0697-x (PMC6329166; doi:10.1186/s12903-018-0697-x)
Supplement: Supplementary file 1 — Participant questionnaire. This file includes the questionnaire completed by participants in the study (PDF 269 kb) [file 12903_2018_697_MOESM1_ESM.pdf]

Participant Questionnaire

Developing a Cardiovascular Oral Health (CARDIOH) program

Please answer all questions. Most questions require you to put a tick (√) or cross (X) in the box/boxes to indicate your answer. Choose the box/boxes that best match your answer.

***Section A**: Your dental health. Tick all that apply*		
**1. Do you currently have any of the following problems or concerns with your teeth, gums or mouth?**		
1.1 Bleeding gums	No ☐0	Yes ☐1
1.2 Toothache (pain in your teeth)	No ☐0	Yes ☐1
1.3 Cavities (holes in your teeth)	No ☐0	Yes ☐1
1.4 Loose teeth	No ☐0	Yes ☐1
1.5 Sensitivity (E.g. pain with hot or cold)	No ☐0	Yes ☐1
1.6 Teeth that don't look right (crooked or discoloured teeth)	No ☐0	Yes ☐1
1.7 Dry mouth	No ☐0	Yes ☐1
1.8 Other problems (*please specify*):		

2. Do your dental problems affect what you eat?

☐1 Never

☐2 Sometimes

☐3 Often

If yes, please specify how it has affected you: ______________________________

____

***Section B**: Your visits to the dentist*

3. Have you seen a dentist in the last 12 months?

☐0 No ☐1 Yes ⟶ Go to Question 4

If no, when was your last dental visit?

☐1 More than a year to 2 years

☐2 More than 2 years to 5 years

☐3 More than 5 years

☐4 Don't know

4. Where do you most often see the dentist?

- ☐1 Private dental practice
- ☐2 Government dental clinic (including dental hospital)
- ☐3 Clinical operated by health insurance fund
- ☐4 Armed Services / Defence Force clinic
- ☐5 Other site (*Please specify*):______________________________
- ☐6 Don't know

5. The following are reasons why cardiovascular patients may not see a dentist frequently.

Please indicate how true each statement is for you by circling the number corresponding to your response

		Strongly disagree ↓						Strongly agree ↓
5.1	I am nervous or afraid to seek advice from a dental health professional	1	2	3	4	5	6	7
5.2	I am worried about seeing a dental health professional because it could affect my heart condition	1	2	3	4	5	6	7
5.3	I was advised not to seek dental treatment by my health care provider (doctor, nurse etc.)	1	2	3	4	5	6	7
5.4	I do not feel well enough to go and see a dental health professional	1	2	3	4	5	6	7
5.5	I am too busy to go to see a dental health professional	1	2	3	4	5	6	7
5.6	I have difficulty attending the dentist/ dental clinic because transport is an issue for me	1	2	3	4	5	6	7
5.7	I do not see a dental health professional because I have other priorities	1	2	3	4	5	6	7
5.8	I do not see a dental health professional because it costs too much	1	2	3	4	5	6	7
5.9	I do not see a dental health professional because the wait is too long to access the public dental service	1	2	3	4	5	6	7
5.10	I do not see a dental health professional because it is too painful or uncomfortable	1	2	3	4	5	6	7
5.11	I do not see a dental health professional because it is difficult to make an appointment in the public dental service	1	2	3	4	5	6	7

5 .13 Other issues (*Please specify*):

Please place a tick or cross in the numbered box that best reflects your opinion

6. Overall, how would you generally rate your ease of accessing dental care

Extremely Difficult ☐—☐—☐—☐—☐—☐—☐—☐—☐—☐—☐ **Extremely easy**

Other comments:____

____

____

***Section C**: Oral hygiene habits*

7. How often do you brush your teeth and/or dentures?

- ☐1 A few times a week
- ☐2 Less than once a day
- ☐3 Once a day
- ☐4 Twice a day
- ☐5 More than twice a day
- ☐6 Never

8. Which of the following do you use? (*tick all that apply*)

- ☐1 Fluoride toothpaste
- ☐2 Mouthwash
- ☐3 Dental floss or any other oral health aids
- ☐4 Sugar free chewing gum
- ☐5 Fluoride tablets or drops
- ☐6 None

Other products, please indicate what do you use: ______________________________________

____

***Section D**: Perceptions about oral health (the health of the mouth, gums and teeth)*

9. How would you describe the health of your teeth and mouth

- ☐5 Excellent
- ☐4 Very good
- ☐3 Good
- ☐2 Fair
- ☐1 Poor

10. How important is the health of your teeth and mouth to you compared to your overall health?

Not important at all										Extremely important
☐	☐	☐	☐	☐	☐	☐	☐	☐	☐	☐
0	1	2	3	4	5	6	7	8	9	10

***Section E**: Confidence about looking after your oral health. Tick the answer that most apply to you*

11. The following questions relate to your level of confidence managing your oral health:

	Strongly disagree	Disagree	Unsure	Agree	Strongly Agree
11.1 I am confident that I am able to look after my teeth	☐1	☐2	☐3	☐4	☐5
11.2 I am confident that I know when I have a dental problem	☐1	☐2	☐3	☐4	☐5
11.3 I am confident that I know when to seek dental treatment	☐1	☐2	☐3	☐4	☐5

***Section F**: Your beliefs about oral health (health of the mouth, gums and teeth)*

12. Please select an answer for the following statement listed below:

12.1 Flossing should be done daily to clean in between teeth	True ☐0	False ☐1	Don't know ☐2
12.2 People with heart problems should avoid dental treatment	True ☐0	False ☐1	Don't know ☐2
12.3 People with heart problems should only see a dentist when there is an emergency	True ☐0	False ☐1	Don't know ☐2
12.4 Poor oral health may affect an existing heart condition	True ☐0	False ☐1	Don't know ☐2
12.5 People with existing heart problems should visit a dentist regularly for a check up	True ☐0	False ☐1	Don't know ☐2
12.6 Some medications for high blood pressure can cause people to experience dry mouth	True ☐0	False ☐1	Don't know ☐2
12.7 Dry mouth does not increase the risk of dental decay	True ☐0	False ☐1	Don't know ☐2
12.8 Some medications for high blood pressure can affect the sense of taste	True ☐0	False ☐1	Don't know ☐2
12.9 Some heart medications can cause swelling or overgrowth (thickening) of the gums	True ☐0	False ☐1	Don't know ☐2
12.10 The overgrowth of gums can lead to poor oral health	True ☐0	False ☐1	Don't know ☐2
12.11 Bad breath is a sign of gum disease	True ☐0	False ☐1	Don't know ☐2
12.12 Loose teeth is one sign of severe gum disease	True ☐0	False ☐1	Don't know ☐2

Section G: *Information about oral health care in the cardiac setting*

13. Have you received any information about oral health since you have been diagnosed with a heart problem?

☐$_0$ No ⟶ Go to Question 14 ☐$_1$ Yes

(a) If yes, what information did you receive about oral health?

☐$_1$ You were advised to visit a dentist

☐$_2$ Instructions about how to look after your mouth and teeth (e.g. brushing, flossing, mouth rinse, etc.)

☐$_3$ Oral health promotion material such as leaflets, pamphlets, samples or other

☐$_4$ Other advice (*please specify*):____

(b) Who provided you the information ?

Please specify (e.g. doctor, nurse, educators, etc.): ____

(c) When did you receive information about oral health?

Please specify (e.g. before admission to hospital, ward, on discharge, etc.) ______________________

____

Section H: *Oral health services provided by cardiac nurses*

**14.**	**The following questions relate to cardiac nurses:**			
14.1	Do you think cardiac nurses could assist you in identifying oral health problems?	No ☐$_0$	Yes ☐$_1$	Don't know ☐$_2$
14.2	Would you consider oral health advice given by cardiac nurses?	No ☐$_0$	Yes ☐$_1$	Don't know ☐$_2$
14.3	Do you think cardiac nurses have sufficient knowledge about oral health to advise you?	No ☐$_0$	Yes ☐$_1$	Don't know ☐$_2$
14.4	Would you make an appointment to see a dentist if you were provided a dental referral by a cardiac nurse?	No ☐$_0$	Yes ☐$_1$	Don't know ☐$_2$

15. The following are services that could be provided to improve access to dental care for patients with a heart condition

How likely are you to participate in these services if they are provided?

		**Very unlikely**						**Very likely**
15. 1	Cardiac nurses asking questions to find out about your oral health	**1**	**2**	**3**	**4**	**5**	**6**	**7**
15.2	Cardiac nurses offering you dental advice	**1**	**2**	**3**	**4**	**5**	**6**	**7**
15.3	Cardiac nurses providing you with oral health information resources such as leaflets, pamphlets or others	**1**	**2**	**3**	**4**	**5**	**6**	**7**
15.4	Cardiac nurses visually checking your mouth and teeth	**1**	**2**	**3**	**4**	**5**	**6**	**7**
15.5	Cardiac nurses referring you to a dentist	**1**	**2**	**3**	**4**	**5**	**6**	**7**
15.6	Having priority access to public dental service	**1**	**2**	**3**	**4**	**5**	**6**	**7**
15.7	Receiving free vouchers to attend private dental services	**1**	**2**	**3**	**4**	**5**	**6**	**7**

16. When do you think is the best time to have an oral health check by the cardiac nurse?

- ☐1 Before admission to hospital
- ☐2 Sometime during your stay in hospital
- ☐3 Before discharge from hospital
- ☐4 At cardiac rehabilitation
- ☐5 At another time (*please specify*): ____________________

17. When do you think is the best time to receive oral health educational material from cardiac nurses?

- ☐1 Before admission to hospital
- ☐2 Sometime during your time in hospital
- ☐3 Before discharge from hospital
- ☐4 At cardiac rehabilitation
- ☐5 At another time (*please specify*): ____________________

***Section I**: Your social and family support*

**18. The following questions relate to your network and social support to access dental care services**			
18.1	Do you have someone (family or friend) to give you support when you have a dental problem?	☐0 No	☐1 Yes
18.2	Do you have someone (family or friend) to talk about your dental problems if you have any?	☐0 No	☐1 Yes
18.3	Do you have someone (family or friend) who can take you to dental appointments if necessary?	☐0 No	☐1 Yes
18.4	Do you have financial support to see a dentist or have dental treatment if necessary?	☐0 No	☐1 Yes
18.5	Do you have easy access to transport if you need to go to a dental appointment?	☐0 No	☐1 Yes

***Section H**: Finally, some questions about you*

**19. Age at your last birthday**	years
**20. Country of birth**	
**21. Language spoken at home**	
**22. Name of cardiac condition(s) that you have been diagnosed with**	
**23. Name any other medical conditions you have been diagnosed with**	
**24. Number of years since your diagnosis with a cardiac condition**	years
**25. Employment status**	☐0 Not working ☐1 Part-time ☐2 Full-time
**26. Are you currently married or living with a partner?**	☐0 No ☐1 Yes
**27. What is your postcode**	

28. What is your highest educational qualification?

☐1 No formal schooling

☐2 Primary school

☐3 Secondary school

☐4 TAFE

☐5 College or University

29. What is your average annual household income (combined)?

- ☐1 Less than $40,000
- ☐2 $40,000 to $60,000
- ☐2 $60,000 to $80,000
- ☐3 $80,000 to $100,000
- ☐3 $100,000 to $120,000
- ☐4 More than $120,000
- ☐5 Prefer not to answer

**30. Do you have private health insurance**	☐0 No	☐1 Yes	☐2 Don't know
**31. Do you currently have the following:**			
**i) Pensioner concession card**	☐0 No	☐1 Yes	☐2 Don't know
**ii) Health care card**	☐0 No	☐1 Yes	☐2 Don't know
**iii) Department of Veterans Affairs card**	☐0 No	☐1 Yes	☐2 Don't know

Thank you for participating in this survey

For any enquiries contact Paula Sanchez (02) 8738 9352 or Dr Ajesh George (02) 8738 9356.
Email: 11154671@student.westernsydney.uws.edu.au
